# Postpartum Cardiovascular Health in African Women Following Pre‐Eclampsia: A Prospective Cohort Study

**DOI:** 10.1111/1471-0528.18192

**Published:** 2025-04-28

**Authors:** Annettee Nakimuli, Emmy Okello, Annette Kesiiga, Moses Adroma, Jackline Akello, Sheila Nabweyambo, Musa Sekikubo, Imelda Namagembe, Robert Kayesubula, Ronald Galiwango, Brittany Jasper, Ashley Moffett, Catherine E. Aiken, Ian B. Wilkinson, Carmel M. McEniery

**Affiliations:** ^1^ Department of Obstetrics and Gynaecology School of Medicine, Makerere University College of Health Sciences Kampala Uganda; ^2^ Uganda Heart Institute At Mulago Kampala Uganda; ^3^ Kawempe National Referral Hospital Kampala Uganda; ^4^ Mulago Specialized Women and Neonatal Hospital Kampala Uganda; ^5^ Department of Medical Physiology School of Biomedical Sciences, Makerere University College of Health Sciences Kampala Uganda; ^6^ Makerere University Infectious Diseases Institute Kampala Uganda; ^7^ Department of Obstetrics and Gynaecology University of Cambridge, The Rosie Hospital and NIHR Cambridge Biomedical Research Centre Cambridge UK; ^8^ Department of Pathology and Centre for Trophoblast Research University of Cambridge Cambridge UK; ^9^ Division of Experimental Medicine and Immunotherapeutics University of Cambridge, Addenbrookes Hospital Cambridge UK

**Keywords:** Africa, arterial stiffness, cardiovascular disease, hypertension, low‐resource, maternal health, postpartum surveillance, pre‐eclampsia, Uganda

## Abstract

**Objective:**

To investigate the prevalence of hypertension and cardiovascular dysfunction 1 year postpartum in Black African women who experienced pre‐eclampsia in a low‐resource setting in Uganda.

**Design:**

Prospective cohort study.

**Setting:**

Tertiary referral hospital in urban Uganda.

**Population:**

Pregnant women who developed pre‐eclampsia between 2019 and 2021, matched to normotensive controls with maternal and gestational age.

**Methods:**

Sociodemographic, clinical and laboratory data were collected at recruitment and 1 year postpartum. Baseline characteristics and incidence rate ratios were calculated to assess risk factors for developing hypertension. Multivariable conditional Poisson regression adjusted for matched study design was used to analyse outcomes.

**Main Outcome Measures:**

The primary outcome was hypertension (≥ 140/≥ 90 mmHg) at 1 year postpartum. Secondary outcomes included aortic pulse wave velocity, left ventricular mass index, and left ventricular ejection fraction at 1 year postpartum.

**Results:**

At one‐year postpartum, hypertension prevalence was higher among women with pre‐eclampsia than controls (36.4% (96/264) versus 4.5% (12/264); aIRR 1.26, 95% CI 1.16–1.36, *p* < 0.001). Postpartum median aortic pulse wave velocity was increased in women with pre‐eclampsia (6.45 ± 0.76 m/s vs. 5.67 ± 0.22 m/s, *p* < 0.001). Left ventricular mass indexed to body surface area was increased in women with pre‐eclampsia (71.7 ± 19.6 g vs. 76.5 ± 23.2 g, *p* < 0.01). Left ventricular ejection fraction was not influenced by pre‐eclampsia (*p* = 0.35).

**Conclusions:**

In this low‐resource setting, black African women with pre‐eclampsia had increased cardiovascular risk markers at one year postpartum. Over one‐third of women with pre‐eclampsia developed hypertension at one year postpartum, emphasising the need for postpartum blood pressure monitoring and early intervention to mitigate long‐term cardiovascular risk in this high‐risk population.

**Abbreviations**
aIRR: adjusted incidence rate ratio
ALT: alanine aminotransferase
aPWV: Aortic pulse wave velocity
AST: aspartate transaminase
BMI: body mass index
DBP: Diastolic blood pressure
E/A ratio: mitral inflow pattern
HDL: high density lipoprotein
ISVd: interventricular septal thickness in diastole
LAD: left atrial diameter
LDL: low density lipoprotein
LVDD: left ventricular diameter diastole
LVDS: left ventricular diameter systole
LVEF: left ventricular ejection fraction
LVM: left ventricular mass
LVMi: left ventricular mass index
PWd: posterior wall thickness in diastole
RWT: relative wall thickness
SBP: Systolic blood pressure

## 1 Introduction

Pre‐eclampsia is a leading cause of maternal morbidity and mortality globally, but particularly so in sub‐Saharan Africa [1]. In urban Uganda, pre‐eclampsia accounts for a quarter of all maternal deaths [2]. Pre‐eclampsia is also associated with reduced fetal growth and iatrogenic preterm delivery [3], which are major causes of perinatal morbidity [4].

The consequences of pre‐eclampsia may extend beyond pregnancy and the immediate postpartum period [5]. Studies suggest that women who experience pre‐eclampsia are at increased risk of cardiovascular disease later in life [6, 7, 8]; however, the trajectory of cardiovascular risk is not well‐characterised [7, 9, 10] and much of this evidence comes from non‐African populations. As women of African ancestry are at higher risk of pre‐eclampsia [11], it is particularly important to understand the implications of this diagnosis for their future health. The rapidly increasing burden of cardiovascular disease in sub‐Saharan Africa [12] makes finding ways to identify and screen high‐risk individuals an urgent priority. There is currently no global consensus on whether ongoing cardiovascular disease risk in women who have experienced pre‐eclampsia should be monitored or mitigated.

We aimed to investigate the prevalence of surrogate cardiovascular disease endpoints, including hypertension, arterial stiffness, and cardiac function, at 1 year following pregnancy complicated by pre‐eclampsia in a large and well‐characterised cohort of Indigenous Black African women.

## 2 Methods

### 2.1 Study Setting and Population

A prospective cohort study was conducted at Mulago National Referral and Teaching Hospital in Kampala, Uganda. As the largest maternity centre in sub‐Saharan Africa and the main tertiary referral centre for Uganda, Mulago Hospital facilitates over 30 000 births annually (80–100 births per day), making it one of the busiest maternity hospitals worldwide.

### 2.2 Study Design and Outcome Measures

Participants recruited were pregnant women (≥ 20 weeks gestation) with pre‐eclampsia and a normotensive control group into a prospective cohort study from March 2019 to June 2021. The groups were loosely matched 1:1 for maternal age (within bands of 5 years) and gestational age at recruitment (20–28 weeks, 29–33 weeks, 34–37 weeks, ≥ 38 weeks).

Pre‐eclampsia was defined according to context‐appropriate modification of ACOG guidelines from 2013 [13]. Participants were defined as having pre‐eclampsia if they were ≥ 20 weeks of gestation, calculated based on either weeks of amenorrhea or ultrasound scan, and had (i) new onset hypertension (systolic blood pressure (BP) ≥ 140 and/or diastolic BP ≥ 90 mmHg, on more than one occasion at least 4 h apart) and (ii) proteinuria of +1 or more by urine dipstick on at least one occasion. Blood pressure readings were taken with the Omron digital automatic blood pressure monitors. The full study inclusion and exclusion criteria are demonstrated in Table 1.

**TABLE 1:** Inclusion and exclusion criteria for study recruitment.

Inclusion criteria	Exclusion criteria
≥ 20 weeks pregnant assessed by weeks of amenorrhea or ultrasound scan	Women with pre‐existing conditions: Chronic hypertension Diabetes mellitus Chronic renal disease Cardiac disease
Black African, born in Africa, and whose parents were also Black Africans born in Africa	
Informed, written consent to participate in the study	
Pre‐eclampsia group:	
New onset hypertension of BP ≥ 140/≥ 90 mmHg, on more than one occasion at least 4 h apart	
Proteinuria of +1 or more by urine dipstick on at least one occasion	
Normotensive group:	
Normotensive (diastolic BP < 90 mmHg and systolic BP < 140 mmHg) throughout the antenatal period, labour, and the first 24 h postpartum	

Potential participants with pre‐eclampsia were identified and recruited sequentially by research midwives from women admitted to obstetric emergency wards, those awaiting admission, and those in antenatal clinics. Women were recruited either prior to active labour or after delivery for those who were already in active labour. The normotensive control group was recruited in antenatal clinics. Information about the study was given to eligible women and those who agreed to participate gave informed written consent. Participants were asked to contact the research team every subsequent time they came to hospital for antenatal care or delivery.

### 2.3 Study Endpoints (At One‐Year Postpartum)

The primary study endpoint was hypertension (BP ≥ 140/≥ 90 mmHg) at 1 year postpartum. The secondary study endpoints were: aortic pulse wave velocity (aPWV; adjusted for mean arterial pressure), left ventricular diameter (LVD) in systole and diastole, and left ventricular ejection fraction (LVEF).

### 2.4 Sample Size Estimation

The study was powered to detect a twofold difference in the proportion of women in each arm found to be hypertensive at one‐year postpartum, assuming a prevalence of hypertension in women with pre‐eclampsia as ~34%, suggested by our previous work in the same setting [14]. A sample size of 264 exposed and 264 unexposed gives 99.5% power to detect a two‐fold difference between groups at an alpha level of 0.05.

### 2.5 Data Collection

Sociodemographic and clinical data were collected using interviewer‐administered questionnaires at enrolment and one year postpartum. Personal and medical history were self‐reported by women, including characteristics such as age, education level, marital status, religion, alcohol consumption, primiparity, history of infertility (all causes), and relevant family history (diabetes mellitus, pre‐eclampsia, hypertension). Laboratory investigations were performed at enrolment, including urine protein quantification via urine dipstick, random blood sugar, renal function tests (serum creatinine and uric acid), liver function tests (ALT, AST), lipid profiles (HDL, LDL, total cholesterol) and full blood count.

At 1 year postpartum the participants had their urine protein, fasting blood sugar, blood pressure, height, and weight measured. Blood pressure measurements were obtained using OMRON (HEM‐907) digital automated blood pressure monitors in a seated position, taking an average of three readings. Hypertension was defined as either a blood pressure of ≥ 140/90 mmHg in the absence of antihypertensive medication.

Transthoracic echocardiography was performed to assess the structure and function of the left ventricle, which was carried out by two specialist‐trained nurses under the supervision of a consultant cardiologist. All examinations were conducted with participants in the left lateral decubitus position after 10 min of rest. Images were acquired by using a standard ultrasound probe placed on the patient's chest to obtain two‐dimensional, M‐mode, and Doppler images of the heart. The following echocardiographic end‐points were measured: left ventricular ejection fraction (LVEF), left ventricular diameter in systole (LVDS) and diastole (LVDD) respectively, left atrial diameter (LAD), interventricular septal thickness in diastole (ISVd), posterior wall thickness in diastole (PWd), mitral inflow pattern (E/A ratio). Derived parameters were calculated from raw echocardiographic data according to the equations in Supplementary Participants with abnormal echocardiogram results were referred to the local cardiology service for further assessment. A VICORDER device was used as described previously [15] to obtain a non‐invasive measure of aortic pulse wave velocity (aPWV), which is a robust indicator of aortic stiffness and independently associated with cardiovascular mortality [16].

All research staff received training on the study processes and measurements before the study began, then monthly for the first 3 months, and on a quarterly basis thereafter. All devices were calibrated and maintained as per manufacturers' recommendations. Standard operating procedures for each research visit were developed and signed off before the first participant was recruited.

To maximise follow‐up rates, we used various strategies including monthly phone calls for updates on participant wellbeing and to remind them of upcoming visits. We also offered fetal growth monitoring, infant immunisation, and feeding advice for infants, and breast and cervical cancer screening for mothers at the 1 year visit.

### 2.6 Data Management

The completed questionnaires were checked for completeness and accuracy. Double data entry was performed using access databases. A confidential register of the details for each participant was maintained and was only accessible to the research assistants and the principal investigator (PI). All other data were pseudonymised by unique study identifier only.

### 2.7 Statistical Analysis

All analyses were conducted according to a pre‐specified analysis plan that was agreed by the PI and study statistician prior to recruitment. The study adhered to the STROBE guidelines for reporting observational studies.

The proportion of women with hypertension at 1 year postpartum in pregnancies affected by pre‐eclampsia compared with pregnancies not affected by pre‐eclampsia was the pre‐specified primary outcome. Two‐sided 95% confidence intervals (CI) for the difference in proportion between the groups were calculated.

Women recruited into the pre‐eclampsia and control groups were matched by gestational age and maternal age at recruitment. Due to the higher‐than‐anticipated loss to follow‐up during the study (attributable to the COVID‐19 pandemic), the women who remained in the study were re‐matched at 1 year, and those who had an appropriate match were retained, leaving 264 complete pairs (*n* = 528 individuals) for analysis. 12.6% (128/1016) of returning participants from the original cohort could not be appropriately matched. Baseline characteristics of the participants in each group were compared using chi‐squared or Fisher's exact tests for categorical data, where appropriate, or Student's *t*‐test for numerical data. At 1 year follow‐up, incidence rate ratios were calculated to assess risk factors for developing hypertension.

We employed conditional Poisson regression due to our matched study design, where controls were paired with cases based on maternal age and gestational age. Our co‐variate selection strategy for multivariable modelling followed a two‐step process: we included variables that showed a potential association (*p* < 0.2) in univariable analyses, as well as established risk factors for hypertension from the literature. These variables were included in a multivariable conditional Poisson regression model to assess their independent associations with hypertension development, whilst accounting for the matched nature of our data.

Transthoracic echocardiographic parameters were expressed as medians and interquartile ranges (IQR) of each group, and analysed using paired and unpaired Wilcoxon rank sum tests. Linear regression models were used to correct aPWV for mean arterial pressure, expressed as adjusted mean ± 95% CI. Paired analysis of aPWV was performed using random‐effects models, with fixed effects for pre‐eclampsia status and mean arterial pressure.

All analyses were conducted using R for statistical computing (version 4.3 [17]). *p* values were considered statistically significant at < 0.05.

### 2.8 Ethics Statement

The study was approved by the School of Medicine Research and Ethics Committee at Makerere University (SOMREC; Ref 2018‐080), the Uganda National Council for Science and Technology (UNCST; Ref HS 2442). All participants gave informed consent to participate. Permission was obtained from the ethics committees to study emancipated minors (pregnant women below 18 years of age) and to obtain third party consent for participants who were too sick to consent. The written third party consent was obtained from either the next of kin or the patient's main attendant in the hospital. If the patient gained capacity during the course of the study, written consent was sought from her directly and this superseded the earlier third party consent. Withdrawal from the study did not influence clinical care, which is provided free to all women in the study setting. Participants received a modest transport refund and compensation for their time at the follow‐up visits.

Women with human immunodeficiency (HIV) are routinely provided with free clinical care in appropriate clinics, and the study team ensured they had access to these clinics. Women diagnosed with pre‐eclampsia were managed according to local clinical protocols. Any participant with newly diagnosed hypertension was referred for appropriate clinical care. Patient involvement from pregnant women in the study setting was sought to ensure that the study protocol and incentives were acceptable to the relevant patient group.

## 3 Results

**FIGURE 1:**
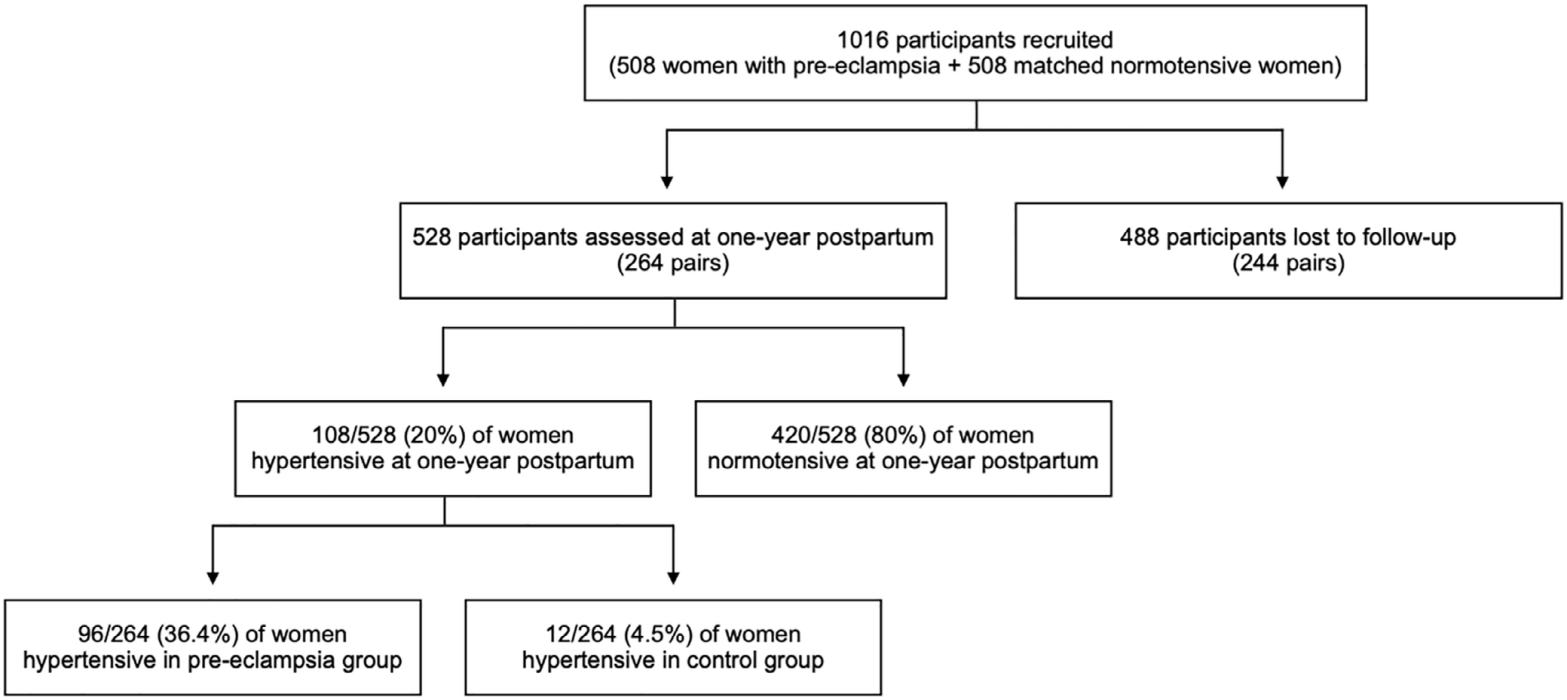
Study cohort diagram. Flow diagram showing numbers of participants in each group at each study stage.

At 1 year postpartum, 528 women were included in the analytic cohort (Figure 1). There were no differences between the women who experienced pre‐eclampsia compared with controls at baseline in terms of their sociodemographic characteristics (Table 2). Women who experienced pre‐eclampsia were more likely to have family histories of pre‐eclampsia (*p* < 0.001) and hypertension (*p* < 0.001) compared with control women (Table 2). The sociodemographic characteristics of women who attended follow‐up at 1 year versus those who did not are shown in Table S1. There were no significant differences in blood pressure metrics between women who attended and those who did not in either the pre‐eclampsia or control groups, and therefore no evidence of attrition bias in terms of the primary study outcome.

**TABLE 2:** Comparison of maternal characteristics of women with and without pre‐eclampsia in Uganda. Numerical data is shown as median ± IQR.

Characteristic		Pre‐eclampsia *n* = 264	No pre‐eclampsia, *n* = 264	*p*
Maternal age (years)		26 (22–31)	26 (22–31)	> 0.9
Maternal BMI		27.4 (23.8–32.5)	26.1 (22.9–30.2)	0.001**
Days of amenorrhea at delivery		259 (234.8–273.0)	283 (275–291)	< 0.001***
Religious affiliation	Catholic Protestant Muslim Born again Other	69 (26.1) 70 (26.5) 69 (26.7) 48 (18.2) 8 (3.1)	73 (27.6) 72 (27.3) 55 (20.8) 54 (20.5) 10 (3.8)	0.6
Maternal education	None or primary Secondary Tertiary/University	65 (24.7) 134 (50.8) 65 (24.6)	58 (22.1) 163 (61.7) 43 (16.3)	0.07
Marital status	Married Unmarried	228 (86.4) 36 (13.6)	236 (89.4) 28 (10.6)	0.3
Alcohol in Pregnancy	No Yes	252 (95.5) 12 (4.5)	254 (96.2) 10 (3.8)	0.8
HIV status	Negative Positive	257 (97.3) 7 (2.7)	253 (95.8) 11 (4.2)	0.5
Family history of diabetes mellitus	No Yes	209 (79.2) 55 (20.8)	224 (84.8) 40 (15.2)	0.11
Family history of pre‐eclampsia	No Yes	248 (93.9) 16 (6.1)	262 (99.2) 2 (0.8)	0.001***
Family history of hypertension	No Yes	154 (58.3) 110 (41.7)	193 (73.1) 71 (26.9)	< 0.001***
Primiparity	No Yes	167 (63.3) 97 (36.7)	182 (68.9) 82 (31.1)	0.2
Pregnancy outcome	Livebirth Fresh stillbirth	217 (82.2) 11 (4.2)	256 (97.0) 2 (0.8)	< 0.001***
	Macerated stillbirth Early neonatal death Unknown	16 (6.0) 5 (1.9) 15 (5.7)	0 (0) 0 (0) 6 (2.2)	
Birth weight centile		24.0 (5.9–57.6)	50.3 (16.0–76.5)	< 0.001***
History of infertility	No Yes	254 (96.2) 10 (3.8)	256 (97.0) 8 (3.0)	0.2
Antenatal clinic attendance	Yes No	256 (97.0) 8 (3.0)	264 (100) 0 (0)	0.007**
Systolic BP at recruitment (mmHg)		176 (164–188)	123 (114–130)	< 0.001***
Diastolic BP at recruitment (mmHg)		117 (107–127)	77 (70–81)	< 0.001***

** *p* < 0.01.

*** *p* < 0.001.

**FIGURE 2:**
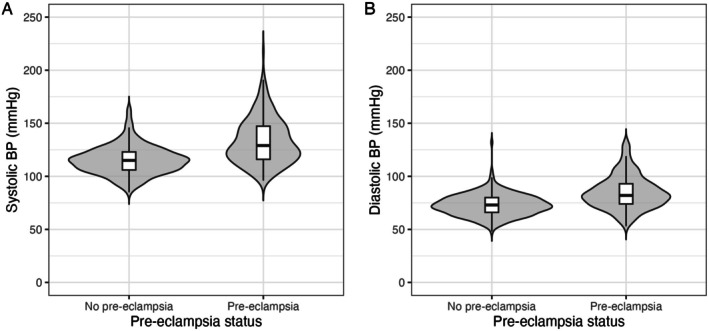
Blood pressures in women who had experienced pre‐eclampsia versus no pre‐eclampsia.

At 1 year postpartum, women who had experienced pre‐eclampsia had higher systolic and diastolic blood pressures than the control group (Figure 2). Women who experienced pre‐eclampsia had a greater risk of having hypertension than women who were normotensive in pregnancy: 96/264 (36.4%) versus 12/264 (4.5%), *p* < 0.001.

In keeping with their diagnosis, women with pre‐eclampsia had higher blood pressure at recruitment (Table 2) and all had proteinuria (Table S2). Women with pre‐eclampsia delivered on average 2 to 3 weeks earlier than the control group despite matching for gestational age at recruitment (259 vs. 283 days of amenorrhea; *p* < 0.001), and their babies had significantly lower birth weights (Table 2). Women with pre‐eclampsia had statistically significant differences in baseline median laboratory parameters compared with control women, in particular higher creatinine, lower platelets, and higher liver enzymes (Table S2).

Characteristics associated with increased risk of hypertension at 1 year postpartum in unadjusted analyses included older maternal age, lower gestational age at delivery, lower birthweight, and adverse fetal outcome (Table S3). There were also statistically significant associations between laboratory markers and increased risk of hypertension (Table S3). HIV positive status was associated with a reduced risk of hypertension at 1 year postpartum (Table S3). In adjusted analysis, increased hypertension risk was associated with pre‐eclampsia (aIRR 1.26, 95% CI 1.16–1.36). Decreases in hypertension risk were associated with HIV positive status and experiencing fresh stillbirth (Table 3). A sensitivity analysis without matching was performed, confirming that pre‐eclampsia remained the most significant measured risk factor for hypertension at one‐year postpartum (Table S4).

**TABLE 3:** Association between maternal characteristics and incidence risk ratio (IRR) of hypertension at one‐year postpartum (multivariate matched analysis).

Characteristic		IIR	95% CI	*p*
No Pre‐eclampsia		*Ref*		
Pre‐eclampsia		1.31	1.22–1.39	< 0.001***
Maternal age (years)		1.01	0.99–1.04	0.24
Maternal BMI		1.00	0.99–1.01	0.77
Maternal education	Primary Secondary Tertiary/University	*Ref* 1.03 1.01	0.93–1.14 0.90–1.14	0.60 0.82
Alcohol in pregnancy	No Yes	*Ref* 1.15	0.96–1.38	0.12
HIV status	Negative Positive	*Ref* 0.81	0.66–1.01	0.06
Birth weight centile		0.99	0.90–1.00	0.22
Pregnancy outcome	Livebirth Fresh stillbirth Macerated stillbirth Early neonatal death	*Ref* 0.70 1.08 1.17	0.46–1.06 0.86–1.36 0.85–1.61	0.10 0.79 0.70
Blood tests taken at one‐year postpartum				
Cholesterol				
Total cholesterol (mmol/L)		1.02	0.96–1.06	0.47
LDL cholesterol (mmol/L)		0.96	0.88–1.04	0.35
HDL cholesterol (mmol/L)		0.98	0.89–1.07	0.69
Full blood count and liver function tests				
Haemoglobin (g/dL)		1.00	0.98–1.02	0.89
Total leucocyte count (x10^3^/μL)		1.00	0.93–1.07	0.34
Neutrophil count (x10^3^/μL)		1.00	0.93–1.08	0.31
Lymphocyte count (x10^3^/μL)		0.99	0.92–1.07	0.88
Aspartate transferase (U/L)		1.00	1.00–1.00	0.11
Alanine transferase (U/L)		1.00	1.00–1.00	0.13

*** *p* < 0.001.

Women who experienced pre‐eclampsia had increased aPWV compared with women who were normotensive in pregnancy at one year postpartum (Table 4; Figure S1). Both systolic and diastolic LVDs were increased in women who had experienced pre‐eclampsia compared with women who were normotensive in pregnancy, although the absolute differences in median diameters were small (Table 4). There was no difference between groups in LVEF, E/A ratio, PWd, IVSd, or RWT (Table 4). The LAD tended to be higher in women who had experienced pre‐eclampsia, but this was not statistically significant (*p* = 0.07 in unpaired, *p* = 0.12 in paired analysis; Table 4). Women who had experienced pre‐eclampsia had higher left ventricular mass, both in terms of absolute mass (*p* = 0.007) and when indexed to BSA (*p* = 0.01; Table 4; Table S5).

**TABLE 4:** Cardiovascular disease surrogate markers at one year postpartum by pre‐eclampsia status. Numerical data is shown as median ± IQR.

Characteristic	Pre‐eclampsia, *N* = 259	No pre‐eclampsia, *N* = 259	Unpaired *p*	Paired *p*
Aortic pulse wave velocity (m/s)	6.45 (5.68–7.21)	5.67 (5.48–5.88)	< 0.001	< 0.001***
LV diastolic diameter (cm)	4.40 (4.20–4.80)	4.40 (4.10–4.60)	0.047	0.021*
LV systolic diameter (cm)	2.79 (2.50–3.00)	2.68 (2.50–2.90)	0.035	0.029*
Ejection fraction (LVEF %)	69 (64–73)	70 (65–74)	0.25	0.35
Left atrial diameter (cm)	3.0 (2.7–3.3)	2.9 (2.7–3.2)	0.07	0.12
Mitral inflow (E/A ratio)	1.3 (1.0–1.4)	1.0 (1.0–1.2)	0.22	0.22
Septal thickness (IVSd)	0.9 (0.8–1.0)	0.9 (0.8–0.9)	0.14	0.13
Posterior wall thickness (PWd)	0.9 (0.8–1.0)	0.9 (0.8–0.9)	0.10	0.32
Relative wall thickness (RWT)	0.4 (0.35–0.45)	0.4 (0.36–0.44)	0.62	0.62
Left ventricular mass (LVM)	125.0 (105.3–150.8)	118.6 (104.5–137.7)	0.01	0.007**
Left ventricular mass index (LVMi)	76.5 (65.4–90.9)	71.7 (62.8–85.0)	0.006	0.01*

* *p* < 0.05.

** *p* < 0.01.

*** *p* < 0.001.

## 4 Discussion

### 4.1 Main Findings

Women in this low‐resource urban setting who experienced pre‐eclampsia during pregnancy had a greater than one‐in‐three chance of being hypertensive at 1 year postpartum. Women who had experienced pre‐eclampsia also had increased arterial stiffness and increased left ventricular mass index at 1 year postpartum compared with women who were normotensive during pregnancy. These findings highlight a concerning future high risk of cardiovascular events in Black African women after experiencing pre‐eclampsia. This is consistent with evidence from other populations where pre‐eclampsia is associated with long‐term cardiovascular disease risk, including stroke and myocardial infarction, over a 15–20 year follow‐up [18]. Notably, even though the women in this cohort were generally healthy and young with a median age of 26 years, without pre‐existing conditions such as chronic hypertension, diabetes mellitus, renal or cardiac disease, 34% of those who experienced pre‐eclampsia were hypertensive at 1 year postpartum.

### 4.2 Strengths and Limitations

Our study design offers several significant strengths. This study recruited a large and well‐characterised cohort of Indigenous African women from a low‐resource setting in urban Uganda. Robust and standardised measurements of validated surrogate cardiovascular endpoints [16, 19] were made, drawing on our previous experience of conducting cardiovascular research in this setting [14], allowing for high accuracy and relevance in understanding cardiovascular risk after pre‐eclampsia.

There are also several limitations to our study. One year postpartum follow‐up rate was 52%, which introduces the possibility of attrition bias in our results. COVID‐19 restrictions throughout Uganda from March 2020 to May 2022 significantly impacted follow‐up, as participants faced difficulties travelling to the centre for follow‐up visits and hesitancy to attend a hospital setting with young infants. Despite this, an analysis as shown in Table S1 indicates that baseline characteristics between participants who attended follow‐up and those who were lost to follow‐up did not differ significantly, lessening the potential impact of attrition bias in the study findings. Reliance on self‐reported questionnaires for collecting personal and medical history data introduces the possibility of social desirability or recall bias.

### 4.3 Interpretation (In Light of Other Evidence)

Our results demonstrate a significant burden of early postpartum hypertension and imply an elevated long‐term risk of cardiovascular disease for this young, urban African population, with implications for both immediate healthcare needs and life‐course health. At a median age of 26, many women in this cohort are likely to have future pregnancies, often with relatively short inter‐pregnancy intervals [20]. Data from a recent meta‐analysis suggests that the relative risk of experiencing a cardiovascular event is approximately doubled in individuals who are hypertensive in young adulthood compared to the general population [20]. The finding that over one‐third of women develop hypertension within just 1 year of a pre‐eclamptic pregnancy highlights an urgent need for structured postpartum follow‐up in this high‐risk group. Given the resource constraints in urban Ugandan primary healthcare settings, innovative approaches to blood pressure monitoring and management may be needed, such as community health worker programmes or mobile health initiatives [21, 22].

Evidence from non‐African cohorts shows that increased arterial stiffness, either resulting from or unmasked by a previous pregnancy, is a risk factor for poor placentation [23, 24] and poor maternal cardiovascular adaptation to subsequent pregnancies [25, 26]. This predisposes both the mother and baby to high risk of adverse outcomes in future pregnancies. These findings have particular significance for progress towards Sustainable Development Goal 3, which aims to reduce maternal mortality and non‐communicable disease burden globally [27]. In low‐ and middle‐income countries, where cardiovascular disease rates are rising rapidly and health systems are often stretched, the combination of pregnancy‐related cardiovascular risk and limited healthcare resources presents a critical challenge to achieving these global health targets. Early identification and management of hypertension could potentially modify these risks, making the immediate postpartum period a critical window for early intervention. In light of these risks, women with pre‐eclampsia may benefit from standardised postpartum blood pressure monitoring, with clear referral pathways when hypertension is detected. However, this would require clinical infrastructure and compete for resource allocation in busy, low‐resource settings. Further, clinicians should consider treating African women who have previously experienced pre‐eclampsia as a high‐risk group, with careful attention to blood pressure monitoring and fetal growth assessment in subsequent pregnancies.

The underlying cause of the elevated cardiovascular markers observed at 1 year postpartum is still uncertain. It remains unclear whether these findings indicate pre‐existing but previously undetected cardiovascular dysfunction, a delayed recovery of the cardiovascular system from the ‘stress test’ of pregnancy, or a pregnancy‐driven permanent change in cardiovascular risk parameters. Smaller Ugandan studies [14, 28] have similarly reported that around 35% of women remain hypertensive at 3 months postpartum after experiencing pre‐eclampsia, aligning with our findings at one‐year. This consistency suggests that a delayed recovery trajectory is less likely to account for our findings, and that a long‐term risk to cardiovascular health has been unmasked or acquired. Further, this reinforces the importance of establishing routine postpartum cardiovascular monitoring programmes that are feasible within the constraints of the local healthcare system.

## 5 Conclusion

Ugandan women who experience pre‐eclampsia have a 36.4% risk of developing hypertension at one year postpartum and display markers indicating an increased risk of subsequent cardiovascular events, particularly increased arterial stiffness and indexed left ventricular mass. These findings highlight the need for systematic postpartum blood pressure monitoring and early intervention strategies that are both effective and feasible within the Ugandan healthcare context. Future research should focus on implementing and evaluating cost‐effective screening and treatment programmes for this high‐risk population.

## Author Contributions

A.N.: conceptualisation, formal analysis, writing – original draft, supervision, funding acquisition, E.O.: investigation, writing – review and editing, A.K.: investigation, writing – review and editing, M.A.: investigation, writing – review and editing, J.A.: investigation, writing – review and editing, S.N.: investigation, writing – review and editing, M.S.: investigation, writing – review and editing, I.N.: investigation, writing – review and editing, R.K.: investigation, writing – review and editing, R.G.: methodology, data curation, formal analysis, writing – review and editing, B.J.: formal analysis, writing – review and editing, A.M.: conceptualisation, writing – review and editing, C.E.A.: formal analysis, writing – original draft, I.B.W.: conceptualisation, writing – review and editing, funding acquisition, C.M.M.: conceptualisation, writing – review and editing, supervision, funding acquisition.

## Ethics Statement

The study was approved by the School of Medicine Research and Ethics Committee at Makerere University (SOMREC; Ref 2018‐080) and the Uganda National Council for Science and Technology (UNCST; Ref HS 2442).

## Consent

All participants gave written informed consent to participate.

## Conflicts of Interest

The authors declare no conflicts of interest.

## Supporting information


Appendix S1.
**Table S1.** Characteristics of participants who attended for follow‐up compared with those who did not. Numerical data: median ± IQR.
**Table S2.** Laboratory parameters of study participants at recruitment by pre‐eclampsia status. Numerical data: median ± IQR.
**Table S3.** Association between participant characteristics and risk of hypertension at 1 year postpartum (unadjusted analysis). IRR: incidence risk ratio. Laboratory values from the point of recruitment.
**Table S4.** Association between participant characteristics and risk of hypertension at 1 year postpartum (multivariate unmatched analysis). IRR (incidence risk ratio) with 95% confidence intervals. Laboratory values from the point of recruitment.
**Table S5.** Derived echocardiography parameter calculations from raw echocardiographic data.
**Figure S1.** Cardiovascular disease surrogate markers at 1 year postpartum by pre‐eclampsia status. (A) Pulse wave velocity, *p* < 0.001. (B) Left ventricular systolic diameter, *p* = 0.03. (C) Left ventricular diastolic diameter, *p* = 0.07 (D) Left ventricular ejection fraction, *p* = 0.015. Blue points represent individuals with pre‐eclampsia, yellow points represent individuals with no pre‐eclampsia. Boxes represent median and IQR for boxes, whiskers represent ±1.5 (IQR).

## Data Availability

The data that support the findings of this study are available from the corresponding author upon reasonable request.
