# Supplementary material for: Postpartum Cardiovascular Health in African Women Following Pre‐Eclampsia: A Prospective Cohort Study
Source: BJOG. 2025 Apr 28;132(9):1319–28. doi: 10.1111/1471-0528.18192 (PMC12232584; doi:10.1111/1471-0528.18192)
Supplement: Supplementary file 1 — Appendix S1. Table S1. Characteristics of participants who attended for follow‐up compared with those who did not. Numerical data: median ± IQR. Table S2. Laboratory parameters of study participants at recruitment by pre‐eclampsia status. Numerical data: median ± IQR. Table S3. Association between participant characteristics and risk of hypertension at 1 year postpartum (unadjusted analysis). IRR: incidence risk ratio. Laboratory values from the point of recruitment. Table S4. Association between participant characteristics and risk of hypertension at 1 year postpartum (multivariate unmatched analysis). IRR (incidence risk ratio) with 95% confidence intervals. Laboratory values from the point of recruitment. Table S5. Derived echocardiography parameter calculations from raw echocardiographic data. Figure S1. Cardiovascular disease surrogate markers at 1 year postpartum by pre‐eclampsia status. (A) Pulse wave velocity, p < 0.001. (B) Left ventricular systolic diameter, p = 0.03. (C) Left ventricular diastolic diameter, p = 0.07 (D) Left ventricular ejection fraction, p = 0.015. Blue points represent individuals with pre‐eclampsia, yellow points represent individuals with no pre‐eclampsia. Boxes represent median and IQR for boxes, whiskers represent ±1.5 (IQR). [file BJO-132-1319-s001.docx]

**Supplementary material**

**Supplementary Table 1.** Characteristics of participants who attended for follow-up compared with those who did not. Numerical data: median ±IQR.

|  | | **Pre-eclampsia** |  | | **Control** |  | |
| --- | --- | --- | --- | --- | --- | --- | --- |
| **Characteristic** | | **Attended follow-up**  **N=264** | **Not attended**  **N=243** | **P-value** | **Attended follow-up N=264** | **Not Attended**  **N=245** | **P-value** |
| Maternal age (years) | | 26 (22-31) | 27 (22-30) | 0.08 | 27 (22-31) | 27 (22-31) | 0.06 |
| Maternal education | None or primary  Secondary  Tertiary/University | 65 (25)  134 (51)  65 (24) | 65 (27)  129 (53)  49 (20) | 0.33 | 58 (22)  163 (62)  43 (16) | 70 (29)  127 (52)  48 (19) | 0.14 |
| Marital status | Married  Not married | 228 (86)  36 (14) | 212 (87)  31 (13) | 0.96 | 236 (89)  28 (11) | 226 (93)  19 (7) | 0.15 |
| HIV status | Negative  Positive | 257 (97)  7 (3) | 236 (97)  7 (3) | 0.89 | 253 (96)  11 (4) | 237 (97)  8 (3) | 0.60 |
| First pregnancy | No  Yes | 167 (63)  97 (37) | 143 (59)  100 (41) | 0.35 | 182 (69)  83 (31) | 162 (66)  83 (34) | 0.46 |
| Days of amenorrhea at delivery | | 259 (235-273) | 250 (226-268) | <0.01** | 283 (275-291) | 281 (273-289) | 0.07 |
| Birth weight (kg) | | 2.4 (1.8-3.0) | 2.2 (1.6-2.8) | <0.01** | 3.3 (3.0-3.6) | 3.2 (3.0-3.5) | 0.08 |
| Antenatal clinic attendance | Yes  No | 256 (97)  8 (3) | 235 (97)  8 (3) | 0.88 | 264 (100)  0 (0) | 245 (100)  0 (0) | 1.00 |
| Systolic BP at recruitment (mmHg) | | 168 (158-182) | 170 (158-184) | 0.35 | 112 (106-121) | 114 (105-123) | 0.78 |
| Diastolic BP at recruitment (mmHg) | | 111 (102-120) | 111 (104-121) 0.26 | | 70 (64-77) | 70 (63-76) | 0.46 |

**Supplementary Table 2.** Laboratory parameters of study participants at recruitment by pre-eclampsia status. Numerical data: median ±IQR.

| **Characteristic** | | **Total**  **N =528** | **Pre-eclampsia**  **n=264** | **No pre-eclampsia**  **n=264** | **P-value** |
| --- | --- | --- | --- | --- | --- |
| Proteinuria  (urine dipstick) | Neg or trace  1+  2+  3+  4+ | 264 (50.0)  43 (8.1)  85 (16.1)  106 (20.1)  30 (5.7) | 0 (0)  43 (16.3)  85 (32.2)  106 (40.1)  30 (11.4) | 264 (100)  0 (0)  0 (0)  0 (0)  0 (0) | <0.001*** |
| Random blood glucose (mmol/l) | | 5.80 (4.80, 6.60) | 6.10 (5.00, 7.10) | 5.30 (4.50, 6.20) | <0.001*** |
| Cholesterol | Total cholesterol (mmol/l) | 5.72 (4.88, 6.61) | 5.99 (5.00, 7.09) | 5.49 (4.84, 6.26) | <0.001*** |
|  | LDL cholesterol (mmol/l) | 3.66 (2.80, 4.47) | 3.85 (2.87, 4.85) | 3.38 (2.70, 4.17) | <0.001*** |
|  | HDL cholesterol (mmol/l) | 1.64 (1.34, 1.97) | 1.67 (1.34, 2.06) | 1.62 (1.35, 1.87) | 0.2 |
| Other blood tests | Serum creatinine (µmol/l) | 48 (39, 62) | 61 (48.8, 80.0) | 42 (35.0, 48.2) | < 0.001*** |
|  | Aspartate transferase (U/l) | 18 (14, 26) | 24 (17.4, 37.6) | 16 (13.2, 19.0) | < 0.001*** |
|  | Alanine transferase (U/l) | 10 (8, 14) | 12 (8.9, 21.0) | 9 (6.9, 10.5) | < 0.001*** |
|  | Haemoglobin level (g/dl) | 12.4 (11.50, 13.50) | 12.90 (11.65, 14.10) | 12.10 (11.40, 12.90) | <0.001*** |
|  | Platelet count (x10^3^ /µl) | 202 (163, 253) | 194 (144.8, 259) | 210 (176.5, 252.5) | 0.028* |
|  | Total leucocyte count (x10^3^/µl) | 7.08 (5.92, 8.56) | 7.66 (6.13, 9.62) | 6.76 (5.76, 7.74) | <0.001*** |
|  | Neutrophil count (x10^3^/µl) | 4.76 (3.80, 6.20) | 5.21 (3.84, 7.12) | 4.45 (3.77, 5.47) | <0.001*** |
|  | Lymphocyte count (x10^3^/µl) | 1.65 (1.36, 1.99) | 1.70 (1.31, 2.04) | 1.60 (1.38, 1.93) | 0.4 |

**Supplementary Table 3:** Association between participant characteristics and risk of hypertension at one-year postpartum (unadjusted analysis). IRR: incidence risk ratio. Laboratory values from the point of recruitment.

| **Characteristic** | | **No hypertension**  **n=419** | **Hypertension**  **n=109** | **IRR (95%CI)** | **P-value** |
| --- | --- | --- | --- | --- | --- |
| No pre-eclampsia | | 252 (60%) | 12 (11%) | *Ref* |  |
| Pre-eclampsia | | 167 (40%) | 97 (89%) | 2.23 (1.74 – 2.87) | <0.001*** |
| Maternal age (years) | | 26 (22-31) | 30 (25-34) | 1.02 (1.00 - 1.05) | 0.027** |
| Maternal education | None or primary  Secondary  Tertiary/University | 92 (22%)  241 (58%)  85 (20%) | 30 (28%)  66 (51%)  23 (21%) | *Ref*  1.05 (0.95 - 1.15)  1.02 (0.91 – 1.13) | 0.35  0.79 |
| Marital status | Married | 365 (87.1%) | 99 (90.8%) | *Ref* |  |
|  | Unmarried | 53 (12.6%) | 10 (9.2%) | 0.93 (0.83 - 1.05) | 0.226 |
| Alcohol in pregnancy | No  Yes | 407 (97.1%)  12 (2.9%) | 99 (90.8%)  10 (9.2%) | *Ref*  1.18 (0.99 - 1.42) | 0.062 |
| HIV status | Negative  Positive | 402 (95.9%)  17 (4.1%) | 108 (99.1%)  1 (0.9%) | *Ref*  0.76 (0.62 - 0.93) | 0.009** |
| First pregnancy | No  Yes | 263 (62.8%)  156 (37.2%) | 86 (78.9%)  23 (21.1%) | *Ref*  0.99 (0.89 - 1.10) | 0.826 |
| Birth weight centile | | 36.0 (12.2-72.2) | 28.7 (7.6-65.0) | 0.99 (0.99 - 1.00) | 0.02* |
| Fetal outcome | Live birth  Fresh stillbirth  Macerated stillbirth  Early neonatal death | 384 (94.8%)  10 (2.5%)  9 (2.2%)  2 (0.5%) | 89 (87.2%)  3 (2.9%)  7 (6.9%)  3 (2.9%) | *Ref*   1. (0.79 - 1.26)   1.46 (1.16 - 1.85)  1.60 (1.10 - 2.32) | <0.9  <0.002**  0.013* |
| Cholesterol | Total cholesterol (mmol/l) | 5.67 (4.85-6.55) | 5.87 (5.19-7.02) | 1.04 (1.01 - 1.06) | 0.005** |
|  | LDL cholesterol (mmol/l) | 3.60 (2.74-4.42) | 3.78 (3.00-4.72) | 1.03 (1.00 - 1.06) | 0.035* |
|  | HDL cholesterol (mmol/l) | 1.64 (1.34-1.94) | 1.61 (1.34-2.06) | 1.02 (0.94 - 1.11) | 0.612 |
| Other blood tests | Serum creatinine (µmol/l) | 47 (39-57) | 60 (45-78) | 1.00 (1.00 - 1.01) | <0.001*** |
|  | Haemoglobin (g/dl) | 12.4 (11.4-13.4) | 12.6 (11.9-13.9) | 1.03 (1.01 - 1.06) | 0.002** |
|  | Platelet count (x10^3^/µl) | 203 (163-253) | 200 (161-253) | 1.00 (0.99 – 1.00) | 0.612 |
|  | Total leucocyte count (x10^3^/µl) | 7.01 (5.89-8.25) | 7.68 (6.09-9.62) | 1.02 (1.01 - 1.03) | 0.003** |
|  | Neutrophil count (x10^3^/µl) | 4.68 (3.80-5.87) | 5.04 (3.95-7.11) | 1.02 (1.01 - 1.03) | 0.004** |
|  | Lymphocyte count (x10^3^/µl) | 1.64 (1.36-1.96) | 1.67 (1.35-2.12) | 1.05 (1.00 - 1.10) | 0.065 |
|  | Aspartate transferase (U/l) | 18 (14.3-24.0) | 22 (15.9-34.4) | 1.00 (1:00 - 1.00) | 0.006** |
|  | Alanine transferase (U/l) | 10 (7.6-13.2) | 11 (8.7-18.8) | 1.00 (1.00 - 1.00) | 0.001** |

**Supplementary Table 4**: Association between participant characteristics and risk of hypertension at one-year postpartum (multivariate unmatched analysis). IRR (incidence risk ratio) with 95% confidence intervals. Laboratory values from the point of recruitment.

| **Characteristic** | | **Incidence rate ratio (IRR)** | **95% CI** | **P-value** |
| --- | --- | --- | --- | --- |
| No pre-eclampsia  Pre-eclampsia | | Ref  1.31 | Ref  1.23 - 1.40 | Ref  <0.001*** |
| Maternal age (years) | | 1.01 | 1.00 -1.02 | <0.001*** |
| Maternal BMI | | 0.99 | 0.99-1.00 | 0.27 |
| Maternal education | None or primary  Secondary  Tertiary/University | Ref  1.01  0.98 | Ref  0.94 - 1.07  0.90 - 1.06 | Ref  0.85  0.56 |
| Alcohol in pregnancy | No  Yes | Ref  1.19 | Ref  1.06 - 1.34 | Ref  0.003** |
| HIV status | Negative  Positive | Ref  0.86 | Ref  0.73 - 1.00 | Ref  0.06 |
| Birth weight centile | | 1.00 | 0.90 – 1.00 | 0.62 |
| Pregnancy outcome | Livebirth  Fresh stillbirth  Macerated stillbirth  Early neonatal death | Ref  0.77  1.01  0.89 | Ref  0.56 – 1.00  0.87 – 1.17  0.76 – 1.44 | Ref  0.06  0.11  0.38 |
| Cholesterol | Total cholesterol (mmol/l) | 1.04 | 0.99 – 1.07 | 0.09 |
|  | LDL cholesterol (mmol/l) | 0.95 | 0.90 – 1.01 | 0.09 |
|  | HDL cholesterol (mmol/l) | 0.96 | 0.92 – 1.03 | 0.46 |
| Other blood tests | Haemoglobin (g/dl) | 1.00 | 0.99 – 1.02 | 0.86 |
|  | Total leucocyte count (x10^3^/µl) | 1.04 | 0.98 - 1.09 | 0.27 |
|  | Neutrophil count (x10^3^/µl) | 0.97 | 0.91 – 1.03 | 0.24 |
|  | Lymphocyte count (x10^3^/µl) | 0.98 | 0.92 – 1.04 | 0.49 |
|  | Aspartate transferase (U/l) | 0.99 | 0.99 – 1.00 | 0.06 |
|  | Alanine transferase (U/l) | 1.00 | 0.99 – 1.00 | 0.41 |

**Supplementary Table 5.** Derived echocardiography parameter calculations from raw echocardiographic data.

| **Echo parameter** | **Formula** |
| --- | --- |
| Left ventricular mass | 0.8 x (1.04 x ((Left ventricular diastolic diameter + interventricular septal wall diameter + posterior wall diameter) – Left ventricular diastolic diameter^3^))) + 0.6 |
| Left ventricular mass index | Left ventricular mass / (0.007184 x (Height (cm)^0.725^) x (Weight (kg)^0.425^) |
| Relative wall thickness (RWT) | 2 x Posterior wall diameter / Left ventricular diastolic diameter |


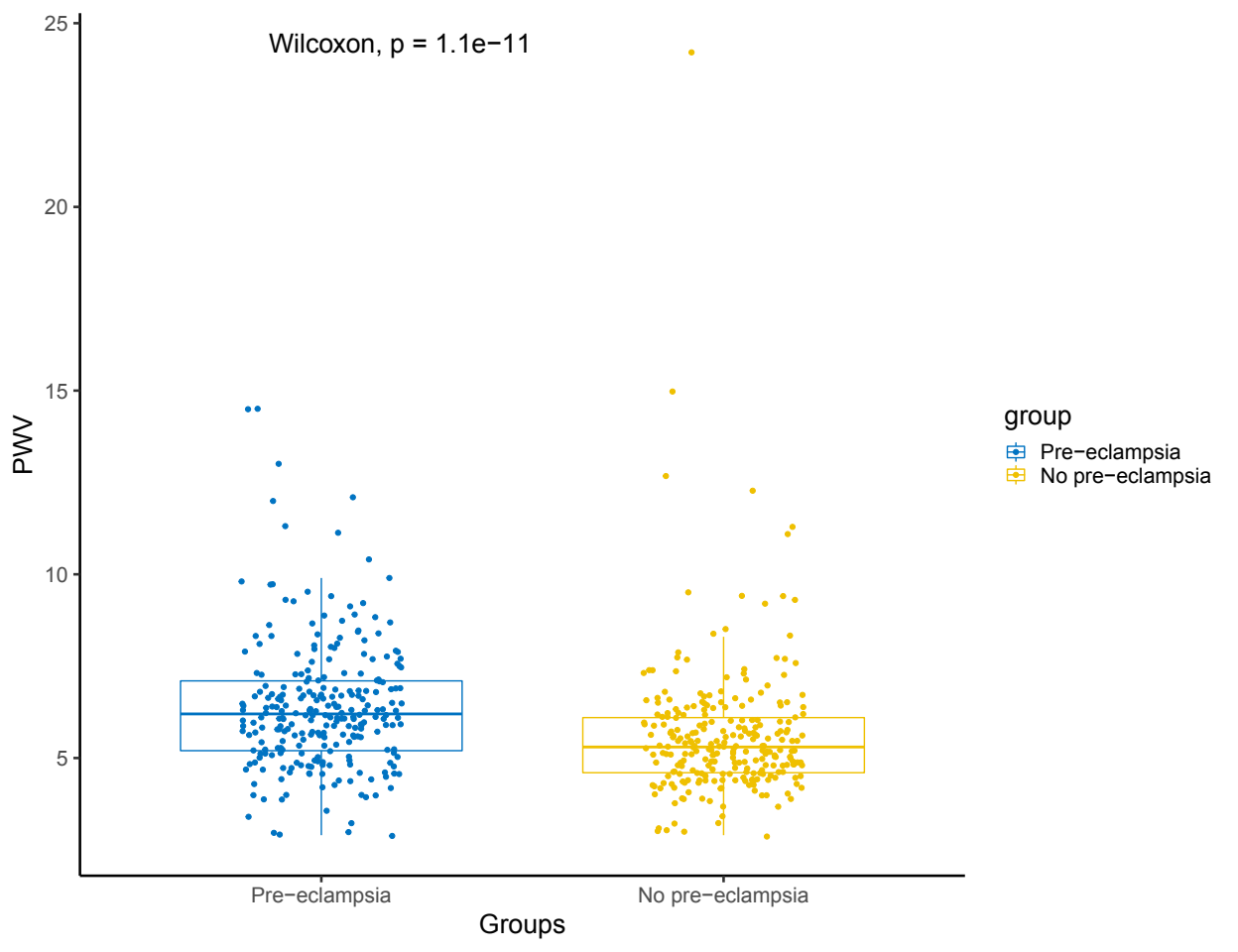

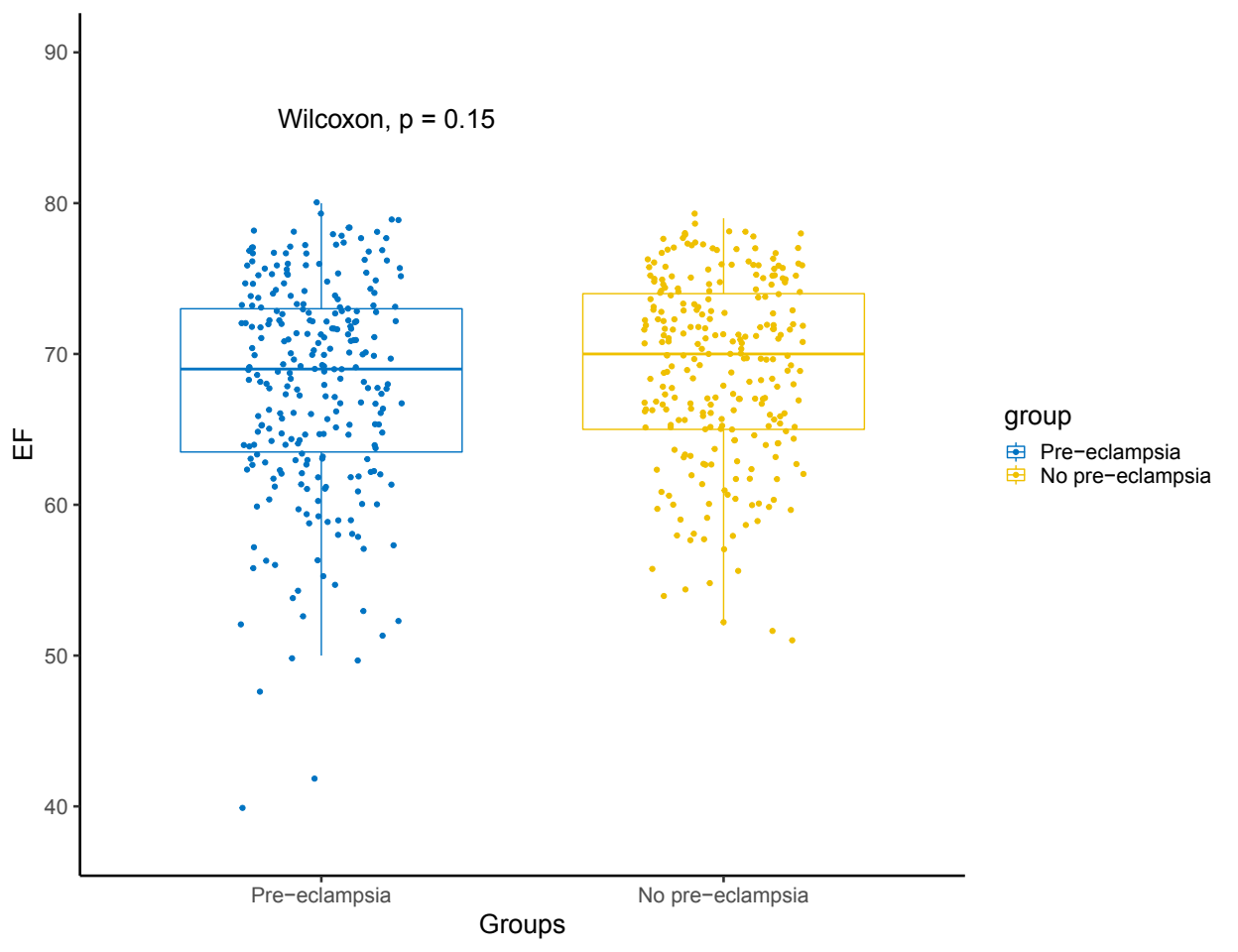

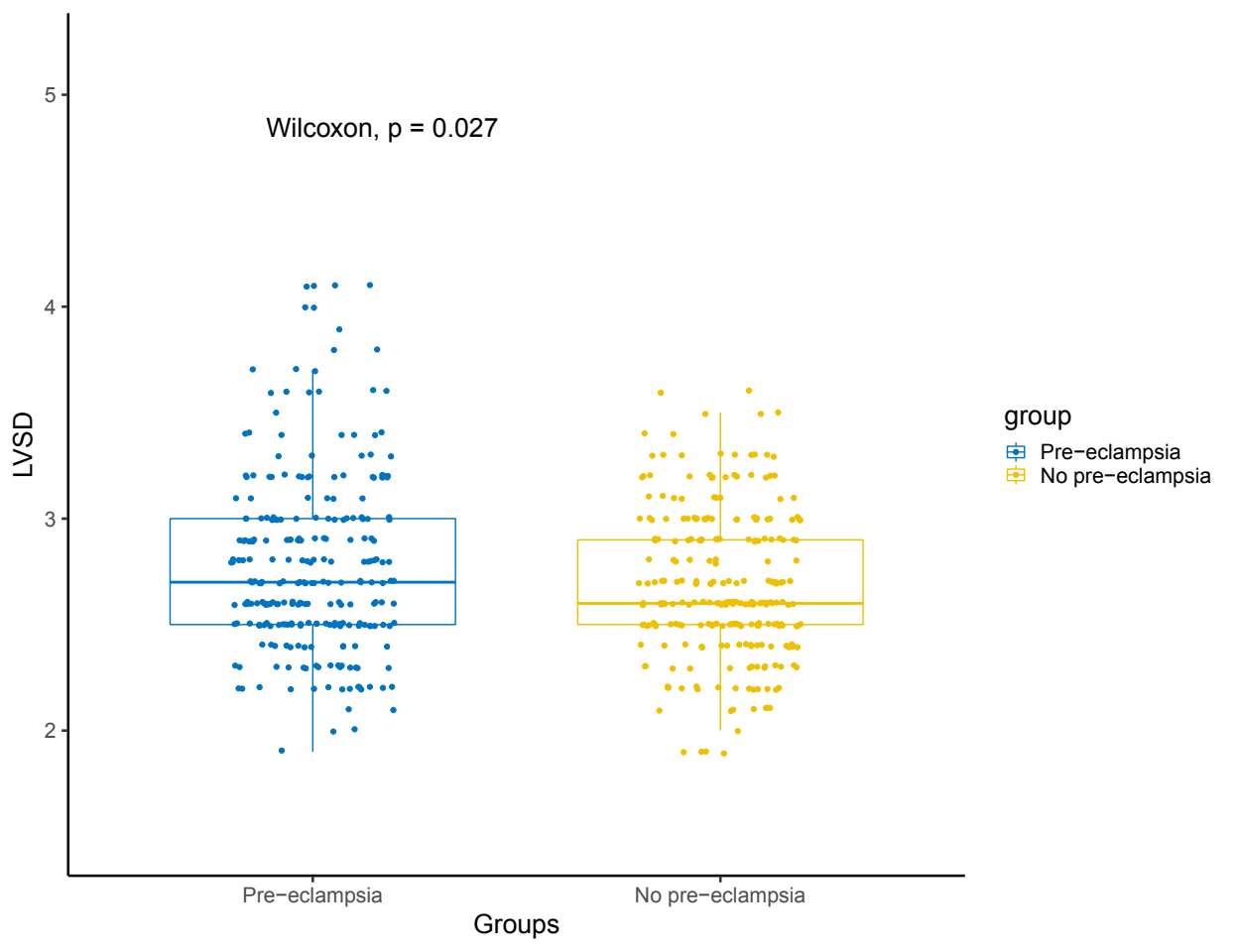

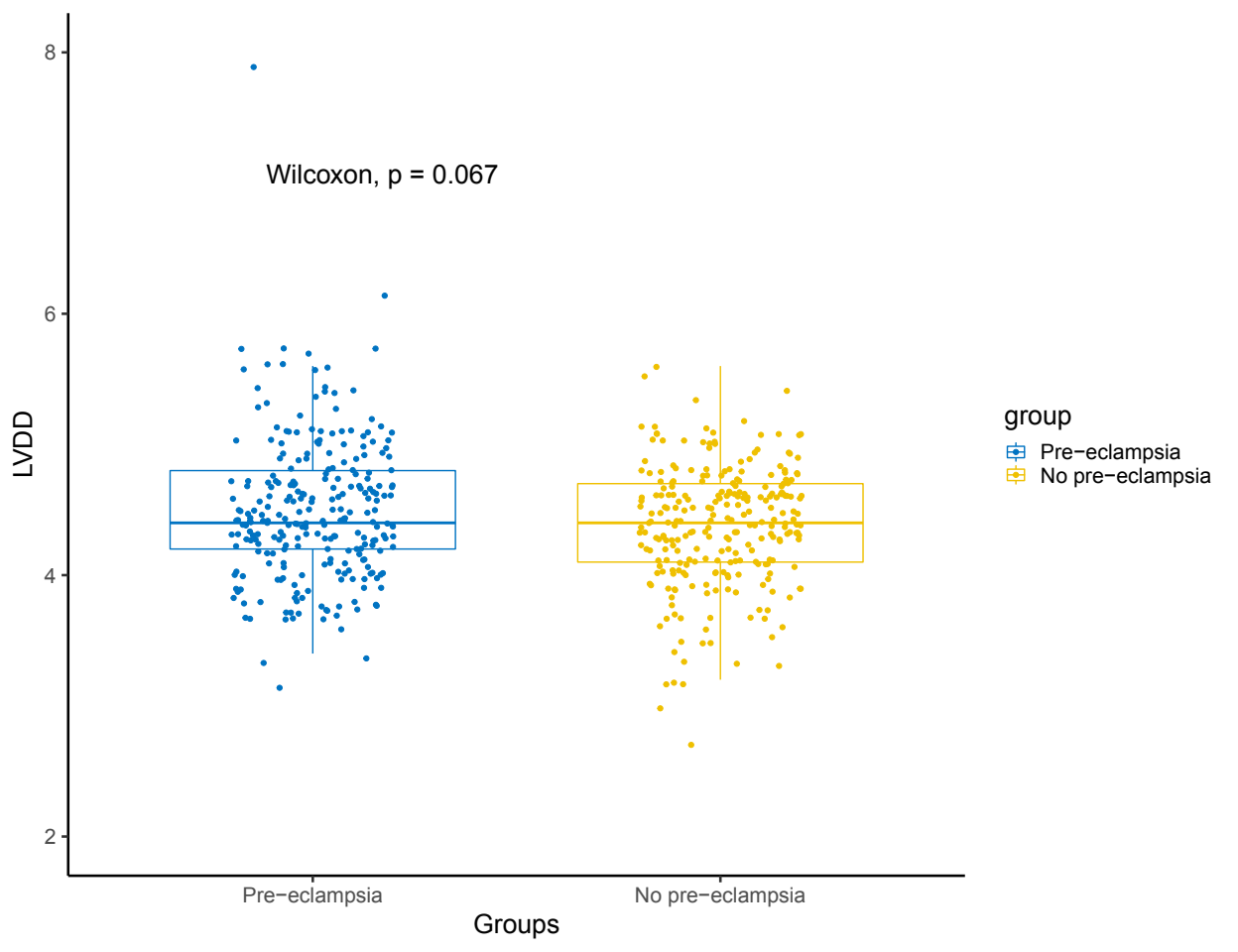


A

B

C

D

**Supplementary Figure 1:** Cardiovascular disease surrogate markers at one-year postpartum by pre-eclampsia status. A) Pulse wave velocity, p<0.001 B) Left ventricular systolic diameter, p=0.03 C) Left ventricular diastolic diameter, p=0.07 D) Left ventricular ejection fraction, p=0.015. Blue points represent individuals with pre-eclampsia, yellow points represent individuals with no pre-eclampsia. Boxes represent median and IQR for boxes, whiskers represent ±1.5(IQR).
